# Comparison efficacy of ultrasound-guided needle release plus corticosteroid injection and mini-open surgery in patients with carpal tunnel syndrome

**DOI:** 10.3389/fneur.2023.1158688

**Published:** 2023-03-29

**Authors:** Zeng Zeng, Nan Lin, Cong-Xian Chen

**Affiliations:** ^1^Cancer Center, Department of Ultrasound Medicine, Zhejiang Provincial People's Hospital, Affiliated People's Hospital, Hangzhou Medical College, Hangzhou, Zhejiang, China; ^2^Zhejiang University School of Medicine, Hangzhou, China; ^3^Plastic Surgery Center, Department of Hand and Reconstruct Surgery, Zhejiang Provincial People's Hospital, Affiliated People's Hospital, Hangzhou Medical College, Hangzhou, Zhejiang, China

**Keywords:** ultrasound, mini-open surgery, carpal tunnel syndrome, transverse carpal ligament, needle release

## Abstract

This retrospective study was to compare clinical outcomes of ultrasound-guided needle release with corticosteroid injection vs. mini-open surgery in patients with carpal tunnel syndrome (CTS). From January 2021 to December 2021, 40 patients (40 wrists) with CTS were analyzed in this study. The diagnosis was based on clinical symptoms, electrophysiological imaging, and ultrasound imaging. A total of 20 wrists were treated with ultrasound-guided needle release plus corticosteroid injection (Group A), and the other 20 wrists were treated with mini-open surgery (Group B). We evaluated the Boston carpal tunnel questionnaire, electrophysiological parameters (distal motor latency, sensory conduction velocity, and sensory nerve action potential of the median nerve), and ultrasound parameters (cross-sectional area, flattening ratio, and the thicknesses of transverse carpal ligament) both before and 3 months after treatment. Total treatment cost, duration of treatment, healing time, and complications were also recorded for the two groups. The Boston carpal tunnel questionnaire and electrophysiological and ultrasound outcomes at preoperatively and 3 months postoperatively had a significant difference for each group (each with *P* < 0.05). There were no complications such as infection, hemorrhage, vascular, nerve, or tendon injuries in both groups. Ultrasound-guided needle release and mini-open surgery are both effective measures in treating CTS patients. Ultrasound-guided needle release plus corticosteroid injection provides smaller incision, less cost, less time of treatment, and faster recovery compared with mini-open surgery. Ultrasound-guided needle release plus corticosteroid injection is better for clinical application.

## Introduction

Carpal tunnel syndrome (CTS) is the most frequent musculoskeletal disorder of compressive neuropathy (1). It is caused by compression of the median nerve within the carpal tunnel between the carpal bones and the transverse carpal ligament (TCL) (2). CTS usually causes sensory (paresthesia and hypoesthesia), pain, sleep disturbance, and motor deficit. It affects ~1–2 men and 4–5 women in every 1,000 persons and always occurs between the age of 40 and 60 years (3). In most cases, CTS is idiopathic (4). Sometimes, diabetes, pregnancy, amyloidosis, and rheumatic synovitis can also cause CTS (4). In essence, CTS is a multifactorial condition (5). CTS is diagnosed by clinical symptoms and physical examination. The Boston carpal tunnel questionnaire, electrophysiological testing, and ultrasonography as supplementary tools can confirm it (6–10). The electrophysiological test of the median nerve is the key point in diagnosing CTS (11). However, when electrophysiological testing is normal in clinically suspected CTS patients, ultrasound imaging seems to be effective in diagnosing (8). Recently, ultrasound was often used for musculoskeletal diseases, especially in peripheral nerve pathologies and median nerve abnormity (12, 13).

There are mainly two types of CTS treatments: surgical and conservative. The choice of treatment depends on the severity of CTS. Conservative treatments are usually for those patients who are in minor and modest circumstances. Conservative treatments include wrist splinting, local corticosteroid injections, laser therapy, and therapeutic ultrasound (14). When conservative ways cannot alleviate CTS symptoms, or the patient's case was severe at the beginning, surgical treatment should be the preferred option (15). Surgery is also a treatment of choice for persistent or progressive CTS patients, and it mainly includes dividing TCL in order to relieve median nerve compression (16). There are five kinds of incision sizes for carpal tunnel release (CTR), namely classic (>4 cm), limited (2–4 cm), mini (1.0–2 cm), percutaneous (0.4–0.6 cm), and ultra-minimally invasive (≤1 mm) (17–22). Smaller incision size shows faster recovery time and lower pain (17, 19). Although endoscopic CTR has superiority compared with classic open CTR (OCTR), it has damage complications to neurovascular structures and tendons (19). Cause mini carpal tunnel release has good outcomes in clinical set-up, and we use mini-incision open surgery when conservative ways cannot alleviate CTS symptoms.

Nowadays, ultrasound-guided corticosteroid injection combined with needle release of the TCL is a new minimally invasive method to treat CTS. Under ultrasound guidance, it is accurate to identify anomalous anatomy, thus avoiding damaging the surrounding tissues of the median nerve. We can observe the median nerve cross-sectional area reduction to demonstrate the effectiveness of corticosteroid injection into the carpal tunnel (23). The purpose of our study was to evaluate the efficacy of surgery and ultrasound-guided corticosteroid injection combined with needle release.

## Materials and methods

### Patients

Our study was approved by the ethical and scientific review board of our hospital. Written informed consent was obtained from every patient. Between January 2021 and December 2021, we included 40 patients (5 men and 35 women) who were diagnosed with CTS in this retrospective study. CTS diagnosis standard was made by clinical history, physical examination, ultrasound, and electrophysiological evaluation. Clinical history included tests of sensibility and muscle strength, questioning symptoms of sensory and thenar atrophy examinations. We checked Phalen's test and Tinel's sign on percussion of the wrist. The criteria of CTS in electrophysiological were median distal motor nerve latency of >4.2 ms (stimulation, 2 cm proximal to the wrist crease) or median sensory nerve conduction velocity of <45 m/s (between the 2 cm proximal to the wrist crease and middle crease of the long finger).

The exclusion criteria were poor general physical condition, pregnancy, carpal fractures, contraindicate for corticosteroid drugs, previous wrist surgery, and previous treatment of CTS. A total of 20 patients (Group A) underwent ultrasound-guided corticosteroid injection combined with needle release, and the other 20 patients (Group B) had mini-open surgery. All patients finished the Boston carpal tunnel questionnaire, ultrasound, and electrophysiological examination before and 3 months after treatment.

### Ultrasound-guided treatment procedures

Ultrasound-guided treatment as an outpatient procedure was routinely performed in the ultrasound interventional room. Ultrasound examinations were performed by a senior ultrasound doctor using the Mindray Resona7 with a 5–12 MHz transducer (Mindray, Shenzhen, China). We set the median nerve in the middle of the screen, and a 5-ml disposable sterile syringe (with a needle) was used for the administration of local anesthesia and release of TCL.

**Figure 1:**
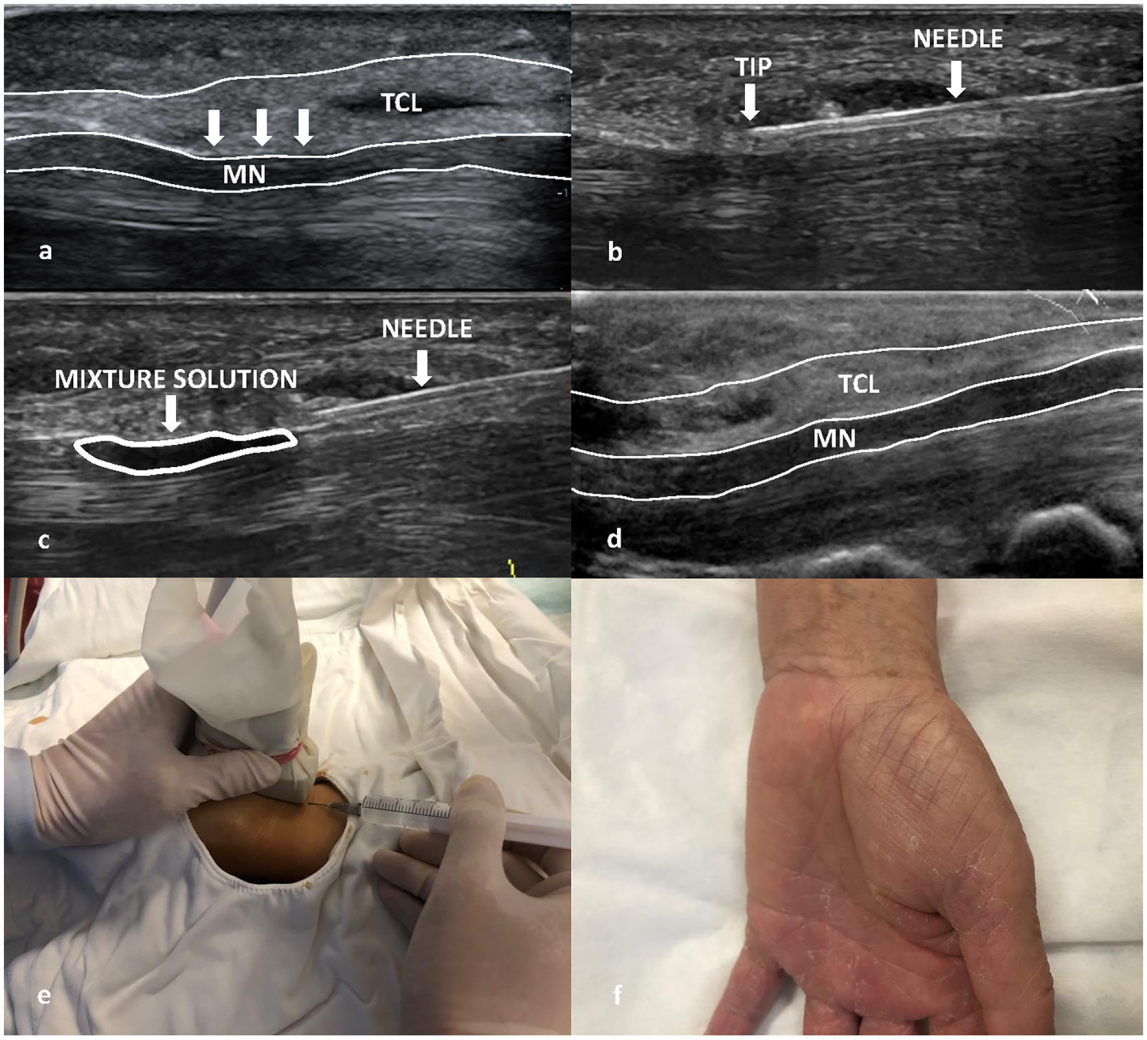
Ultrasound-guided needle release plus corticosteroid injection procedure of a Group A patient. **(a)** The transverse carpal ligament (TCL) was compressed (white arrow) in the ultrasound image before treatment. **(b)** Acupuncture process **(c)** 2 ml mixture solution (white ellipse) which contained 1 ml of 2% lidocaine and 1 ml of betamethasone were injected into the carpal tunnel. **(d)** TCL was normal (no compression) after 3-month follow-up in the ultrasound image. **(e)** Sonographer held the probe covered with surgical gloves with one hand and acupuncture compression of the TCL at the median nerve continuously under the continuous guidance of ultrasound. **(f)** Postoperative image (no scar).

The patient was asked to sit on a chair, and the affected hand was positioned to the side with the palm up in an extended position. All treatment procedures were performed by C.C.X with the probe covered with surgical gloves. An acoustic coupling agent was used on the probe inside the surgical gloves. The patient's skin was disinfected with complex iodine three times from the metacarpophalangeal joint to 5 cm above the carpal canal. The probe was placed on the wrist with the longitudinal section, and then, the long axis of the median nerve was clearly seen. The insertion point is 0.5 cm proximal to the compression point of the median nerve. The direction of the needle entry is from the proximal to the distal end of the median nerve. When inserting the needle, care was taken to avoid stabbing the median nerve, radial artery, and ulnar neurovascular bundle. The needle entry point was wiped with complex iodine cotton balls under ultrasound guidance, and a 4-ml mixture solution which contained 2 ml 2% lidocaine and 2 ml 0.9% sodium chloride (in a ratio of 1:1) was injected. Thereafter, local anesthesia was performed layer by layer on the median nerve surface under the continuous guidance of ultrasound, and acupuncture compression of the TCL at the median nerve was continuously performed from proximal to distal and from shallow to deep. The operation was completed until there was no resistance to the acupuncture of TCL. Under real-time ultrasound guidance, a 2-ml mixture solution containing 1 ml of 2% lidocaine and 1 ml of betamethasone (Schering Pharmaceutical Co., Ltd. in Shanghai, China) (in a ratio of 1:1) was injected, and the mixture solution could be seen diffusing in the carpal tunnel (Figure 1). After the needle was pulled out, the needle path was pressed with hand for 5 min and then pasted with an adhesive bandage at the insertion point. To prevent infection, the wound should be kept dry for 48 h. The whole process lasted for approximately 5–10 min. During the operation, all patients had no pain or discomfort. There were no postoperative complications, and the patient was reexamined 3 months after the operation.

### Mini-open surgery procedures

**Figure 2:**
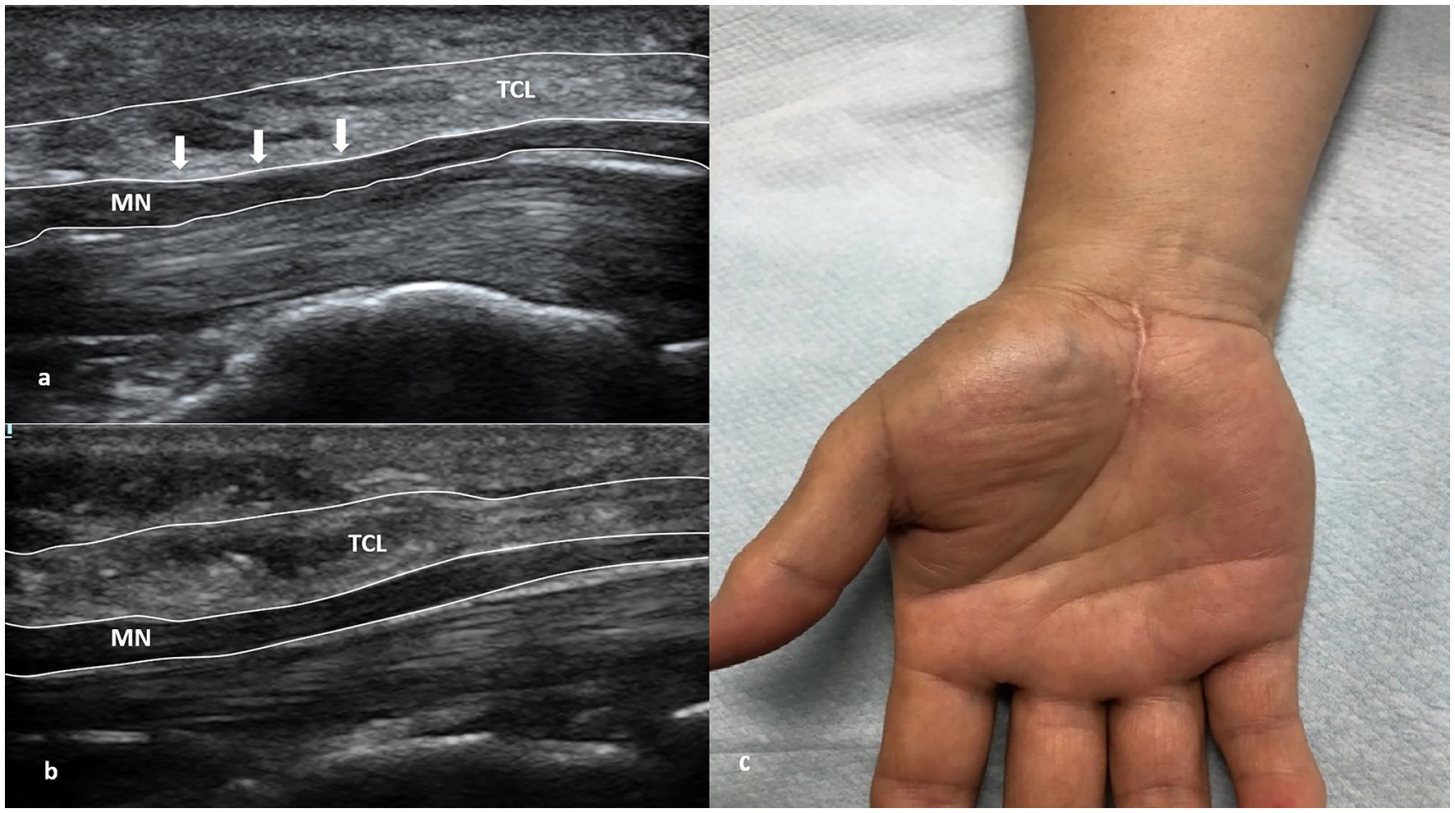
Mini-open surgery of a Group B patient. **(a)** The transverse carpal ligament (TCL) was compressed (white arrow) in the ultrasound image before treatment. **(b)** TCL was normal (no compression) after 3-month follow-up in the ultrasound image. **(c)** Postoperative image (with a 2-cm scar).

The patient was placed in a supine position under general anesthesia. A pneumatic tourniquet was set at 40 kpa pressure, with a total time of 1 h and an interval of 15 min. The size of the incision was ~2 cm, and its shape was longitudinal. The skin and subcutaneous tissues were cut open in turn to expose the carpal canal; the thickened and adherent transverse carpal ligament was cut open; the epineurium and bundle membrane of the nerve were loosened; the nerve bundle decompression was performed; and the wound was rinsed after complete hemostasis (Figure 2). After checking nerve release, it was reclosed layer by layer until it reached the skin.

### Boston carpal tunnel questionnaire, and ultrasonic and electrophysiological evaluations before and after treatment

The Boston carpal tunnel questionnaire is the most widely used to assess symptom severity and functional status. It includes two parts: the Symptom Severity Scale (SSS) and the Functional Status Scale (FSS) (6). SSS has 11 questions, and FSS contains 8 items. According to the mean score, patients are divided into five grades: minimal (0.1–1 point), mild (1.1–2 points), moderate (2.1–3 points), severe (3.1–4 points), and extreme (4.1–5 points).

In ultrasound examinations, the median nerve's flattening ratio (FR) and cross-sectional area (CSA) were measured according to El Miedany and colleagues' grading system (24). Depending on the CSA of the median nerve at the inlet, severity is divided into follows: mild, 10–13 mm^2^; moderate, >13–15 mm^2^; and severe, >15 mm^2^ (25). FR was the ratio of the nerve's transverse axis to the anteroposterior axis. It was assessed at the level of the pisiform bone. The transverse carpal ligament (TCL) was measured on the cross-section at the level of the hamate bone.

In electrophysiological examinations, we recorded the median nerve's distal motor latency (DML), sensory conduction velocity (SCV), and sensory nerve action potential (SNAP). According to recommendations of the American Association of Neuromuscular and Electrodiagnostic Medicine (26) and the American Academy of Orthopedic Surgeon (AAOS) work group (27), CTS severity was classified as follows: negative when all tests had normal findings (both comparative and segmental studies); minimal when abnormal on comparative or segmental tests; mild when normal DML with finger-wrist tract SNCV slowed; moderate when increased DML with finger-wrist tract SNCV slowed; severe when increased DML with a finger-wrist tract absence of the sensory response; and extreme when there is a thenar motor absence of the response.

### Statistical analysis

Statistical analyses were performed using SPSS version 22.0 (SPSS Inc., Chicago, IL, USA). All quantitative data were expressed as mean ± standard deviations (SD). All qualitative data were expressed as numbers and percentages. We used Student's *t*-test to compare quantitative data between groups and Mann–Whitney U-test to compare qualitative data. A *p*-value of < 0.05 was considered to be statistically significant.

## Results

In our study, we included 40 eligible patients (40 wrists) with CTS. There were no significant differences between Group A and Group B with respect to average age, gender, side, and duration of disease (each with *P* > 0.05) (Table 1). For the treatment duration, total treatment cost, and healing time, Group A had significance compared with Group B (each with *P* < 0.05). Group A had a shorter treatment duration, costed less, and had a quicker healing time than Group B. There were no complications such as infection, hemorrhage, vascular, nerve, or tendon injuries in both groups. Patients had no recurrent symptoms after treatment in 3 months.

**Table 1:** Clinical data of two groups.

	**Group A**	**Group B**	***P*-value**
	**(*****n*** = **20 wrists)**	**(*****n*** = **20 wrists)**	
Number of patients	20	20	NA
Gender			0.633
Male	3	2	
Female	17	18	
Side			0.507
Right	12	14	
Left	8	6	
Age (years)	54.05 ± 9.40	53.70 ± 8.22	0.927
Duration of CTS (months)	19.05 ± 15.74	10.50 ± 7.98	0.526
Duration of treatment (minutes)	5.75 ± 1.37	19.00 ± 1.30	0.000^*^
Total treatment cost (dollars)	54.30 ± 3.38	644.55 ± 58.49	0.021^*^
Healing time (months)	1.16 ± 0.14	2.44 ± 0.24	0.000^*^

* *P*-value < 0.05.

Table 2 shows Boston carpal tunnel questionnaire results before and 3 months after the treatment of the two groups. It showed a significant difference for both groups with respect to the Symptom Severity Scale (SSS) and the Functional Status Scale (FSS) before and 3 months after treatment (each with *P* < 0.05). There was no significance regarding SSS and FSS between the two groups 3 months after treatment (*P* = 0.73).

**Table 2:** Boston carpal tunnel questionnaire before and 3 months after treatment for two groups.

	**Group A**			**Group B**			**After treatment Group A vs. Group B**
	**(*****n*** = **20 wrists)**			**(*****n*** = **20 wrists)**			
	**Before**	**After**	***P*** **-value**	**Before**	**After**	***P*** **-value**	***P*** **-value**
**Symptom severity scale (SSS)**							
Normal	0 (0)	9 (45)	0.00^*^	0 (0)	7 (35)	0.00^*^	0.73
Minimal	0 (0)	10 (50)		0 (0)	11 (55)		
Mild	1 (5)	1 (5)		2 (10)	2 (10)		
Moderate	6 (30)	0 (0)		8 (40)	0 (0)		
Severe	10 (50)	0 (0)		8 (40)	0 (0)		
Extreme	3 (15)	0 (0)		2 (10)	0 (0)		
**Functional status scale (FSS)**									
Normal	0 (0)	9 (45)	0.00^*^	0 (0)	7 (35)	0.00^*^	0.73
Minimal	0 (0)	10 (50)		0 (0)	11 (55)		
Mild	1 (5)	1 (5)		2 (10)	2 (10)		
Moderate	6 (30)	0 (0)		8 (40)	0 (0)		
Severe	10 (50)	0 (0)		8 (40)	0 (0)		
Extreme	3 (15)	0 (0)		2 (10)	0 (0)		

* *P*-value < 0.05.

The results of electrophysiological examinations for two groups before and 3 months after treatment are shown in Table 3. There was no significant difference with respect to DML, SCV, and SNAP before treatment between the two groups (each with *P* > 0.05). Both the groups showed significance in their DML, SCV, and SNAP 3 months after treatment which suggested that both ultrasound-guided needle release plus corticosteroid injection and mini-open surgery had effectiveness in treating CTS (each with *P* < 0.05). Furthermore, in terms of DML and SCV 3 months after treatment, there was a significant difference between the two groups (each with *P* < 0.05). It suggested that ultrasound-guided needle release plus corticosteroid injection was superior to mini-open surgery in the matter of DML and SCV.

**Table 3:** Electrophysiologic results before and 3 months after treatment for two groups.

	**Group A**		**Group B**	
	**(*****n*** = **20 wrists)**		**(*****n*** = **20 wrists)**	
**Median nerve**	**Before**	**After**	**Before**	**After**
Distal motor latency (DML, ms)	4.81 ± 1.85	3.50 ± 1.83^+#^	4.80 ± 0.92	3.80 ± 0.89^+^
Sensory conduction velocity (SCV, m/s)	40.65 ± 5.75	51.50 ± 4.51^+#^	38.69 ± 3.09	50.11 ± 6.21^+^
Sensory nerve action potential (SNAP, μV)	7.24 ± 3.30	17.42 ± 7.31^+^	7.10 ± 5.75	19.53 ± 6.76^+^

^+^vs. Before treatment, *P*-value < 0.05.

^#^vs. Group B after treatment, *P*-value < 0.05.

Ultrasound examination results of both groups before and after treatment are shown in Table 4. There was no significant difference with respect to FR, CSA, and TCL before treatment between the two groups (each with *P* > 0.05). There was a significant difference before and 3 months after treatment in the values of FR, CSA, and TCL for both groups (each with *P* < 0.05), which indicated the effectiveness of the two therapeutic approaches. In addition, we found that there was no significant difference between the two groups in the values of FR, CSA, and TCL 3 months after treatment. We performed approximately 1-year follow-up after intervention for our patients. We followed all patients by phone, and the results were that all patients did not develop CTS again.

**Table 4:** Ultrasound results before and 3 months after treatment for two groups.

	**Group A**		**Group B**	
	**(*****n*** = **20 wrists)**		**(*****n*** = **20 wrists)**	
**Median nerve**	**Before**	**After**	**Before**	**After**
Flattening ratio at the level of hamate (FR)	2.89 ± 0.36	2.31 ± 0.20^§^	2.88 ± 0.39	2.34 ± 0.18^§^
Cross-sectional area at the level of pisiform bone (CSA, cm^2^)	0.15 ± 0.02	0.09 ± 0.01^§^	0.14 ± 0.03	0.09 ± 0.02^§^
Transverse carpal ligament-Thickness on the cross-section at the level of hamate bone (TCL, mm)	4.30 ± 0.34	3.45 ± 0.30^§^	4.10 ± 0.43	3.48 ± 0.41^§^

§ vs. Before, *P*-value < 0.05.

## Discussion

CTS is usually diagnosed by illness history and physical examination (28). Electrophysiological examination is often used to confirm it (29). Ultrasound as a new way to diagnose CTS has now been widely used in clinics because it is easy and non-invasive (30). The principle of treating CTS is the decompression of carpal tunnel by opening the median nerve's flattening ratio. This is usually performed by using conventional open surgery (incision size of ~5 cm), mini-open surgery (incision size of ~2 cm), or endoscopy (incision size of ~1.5 cm) (11). Jugovac et al. found that compared with conventional open surgery, mini-open surgery had smaller incisions and returned to work in a shorter time (i.e., 15 days vs. 30 days) (31). In the 1990's, endoscopy came into effect. It is a safe technique that reduces morbidity, postoperative pain, and recovery time (11). However, endoscopy has its own shortcomings with a potential risk of injury to adjacent neurovascular structures (32). Nowadays, a new procedure that uses a needle to release the nerve based on ultrasound as guidance came into use in clinics. Compared with open surgery and endoscopic treatments, ultrasound-guided needle release allows for a fast recovery and easy postoperative care due to almost no wound.

Our research found that CTS was more common in women; we retrospectively studied 40 CTS patients, and 35 of them were female, which was consistent with the study of Tang et al. and Baysal et al. where they found that hand-intensive housewives had a higher incidence of CTS (33, 34). The higher incidence of CTS in women may be due to physiological differences, hormonal changes, and intense housework (35). The mean ages of Group A and B patients were 54.05 ± 9.40 and 53.70 ± 8.22, respectively. CTS incidence peaks for individuals were those people in the age bracket of 40 to 60 years (3).

In our study, we compared the clinical outcomes of ultrasound-guided needle release plus corticosteroid injection and mini-open surgery and found that both groups had significant improvement with respect to BCTQ, electrophysiological, and ultrasound results after treatment. Both groups had no recurrent symptoms or pain syndrome 3 months after treatment. These findings indicated that ultrasound-guided needle release plus corticosteroid injection and mini-open surgery had the same effect in treatment. However, in terms of cost, operation time, recovery time, and recurrence, ultrasound-guided needle release plus corticosteroid injection is better than mini-open surgery. Furthermore, ultrasound-guided needle release plus corticosteroid injection has almost no scar and was deemed to be preferable in terms of cosmesis.

In Group A patients, no complications were reported during or after treatment due to the proper visualization of the carpal tunnel structure under ultrasound guidance. Recent studies demonstrated that corticosteroid injection under ultrasound guidance is better than blind injection (17, 31, 36). The advantage of ultrasound guidance can be clearly listed as follows: (1) The structure can be seen so that the doctor knows the location and reaches the carpal tunnel and injects the medicine without damaging the neighbor tissue; (2) offers a great degree of needle control: the doctor can change the distance of the needle and the median nerve in visualization so that the needle can reach close to the median nerve; and (3) ensures uniform distribution of injection solution around the carpal tunnel and peels the median nerve away from adhesions (37, 38). Studies had shown that the injection of corticosteroids was widely used in the field of pain management because it can reduce pain and swelling. In CTS patients, injecting corticosteroids into the flexor pollicis longus tendon sheath and common flexor sheath can relieve pain and improve functional impairment (39, 40).

During ultrasound-guided needle release plus corticosteroid injection, the following points should be noted: (1) The puncture was carried out along the long axis of the median nerve. During the process of releasing the transverse carpal ligament by acupuncture, attention should be paid to protecting the median nerve deep in the transverse carpal ligament. (2) Since the puncture space of the carpal tunnel is relatively small, appropriate wrist extension can increase the puncture space. The puncture angle should not be too large in the process of puncture, 15°-30° with the skin of the wrist is suitable. (3) Due to the density of the transverse carpal ligament, when the lesion occurs, it will be more tenacious. There will be a sense of resistance during acupuncture, and when the sense of resistance disappears, it indicates that the release is thorough. (4) The puncture should be carried out in the plane so that the needle tip can be observed at all times to avoid injury to the median nerve. (5) Finally, the mixture of lidocaine and betamethasone should not be injected into the transverse carpal ligament during the injection into the carpal canal. When the relationship between the tip and median nerve is not clear, a small amount of normal saline can be injected.

There are many new methods for detecting CTS disease. Shear-wave elastography (SWE) is a new method in the quantitative measurement of tissue stiffness, especially in assessing neuropathy. Several studies have reported the meaning of elastography in diagnosing CTS. Wu et al. investigated the value of both ultrasound and real-time shear wave elastography (SWE) in evaluating the median nerve with CTS and demonstrated that SWE was useful in assessing the median nerve both before and after the operation (41). Recently, some research studies indicated that in CTS patients, blood flow changes of the median nerve can help to confirm. Endo et al. pointed out that using superb microvascular imaging can help clarify CTS patients' blood flow changes (42). They concluded that the diagnostic utility of superb microvascular imaging was superior to conventional color Doppler ultrasound and equivalent to power Doppler ultrasound (42).

There are several limitations to our study. First, the sample size is small, and more data are needed for further study. Second, we only compared ultrasound-guided needle release plus corticosteroid injection with mini-open surgery, and it is still not clear how effective it is compared with endoscopic or other treatment procedures. Furthermore, we only selected 3-month follow-up because some patients returned to work after treatment, and many factors may affect the outcome of follow-up. The long-term effects are still worth watching.

## Conclusion

Both ultrasound-guided needle release plus corticosteroid injection and mini-open surgery had great benefits in the treatment of CTS. Ultrasound-guided needle release plus corticosteroid injection had the advantages of a shorter operation time, quicker recovery time, smaller wounds, and less cost compared with mini-open surgery. According to those advantages, ultrasound-guided needle release plus corticosteroid injection is better for clinical application.

## Data availability statement

The original contributions presented in the study are included in the article/supplementary material, further inquiries can be directed to the corresponding author.

## Ethics statement

The studies involving human participants were reviewed and approved by Zhejiang Provincial People's Hospital. The patients/participants provided their written informed consent to participate in this study.

## Author contributions

ZZ, NL, and C-XC contributed to the study concepts and designed the study. C-XC contributed to the data analysis and interpreted the data. ZZ and NL contributed to the statistical analysis. All authors have read and agreed to the published version of the manuscript.
